# Reproductive Risk Assessment of Bisphenol A and Its Substitutes on Estrogen Receptors (ERs) in Bivalves

**DOI:** 10.3390/ijms26167969

**Published:** 2025-08-18

**Authors:** Weili Guo, Pengyu Zhang, Jianyong Song, Chunnuan Zhang, Ruiyi Xu

**Affiliations:** The Water Environment and Animal Safety Laboratory, Henan University of Science and Technology, Luoyang 471000, China; 15517948598@163.com (W.G.); 13333881963@163.com (P.Z.); 16697782015@163.com (J.S.)

**Keywords:** bisphenol A and its substitutes, bivalves, estrogen receptors, homology model, toxicity

## Abstract

As benthic filter feeders, bivalve mollusks serve as ideal biological indicators. Bisphenol A (BPA) and its substitutes (BPS, BPF, and BPAF) are endocrine disruptors with reproductive toxicity, targeting estrogen receptors (ERs). However, their binding sites and affinity for shellfish ERs remain unclear. This study aims to identify ER binding sites of BPA and its substitutes, compare toxicity via molecular docking, and validate results through exposure experiments. The full-length cDNA of *Corbicula fluminea* ER was cloned using the RACE technique for the first time, the sequence length is 2138bp. Homologous models of LBD sequences from *Danio rerio*, *C. fluminea*, *Azumapecten farreri*, and *Ruditapes philippinarum* ERs were constructed via homology modeling and screened for optimal fit. Hydrogen bonds were observed during the docking process, with interaction sites including Glu-66, Arg-177, and other amino acid residues. Exposure experiments (1, 10, and 100 μg/L) showed an enhancement in ER mRNA expression. Based on the docking energies and results of the exposure experiments, it was concluded that the toxicity of BPA and BPS is similar and greater than that of BPF and BPAF. This study provides data for a reproductive risk assessment and aquatic toxicological monitoring of bisphenols.

## 1. Introduction

Environmental endocrine-disrupting chemicals (EDCs) pose significant threats to human health and ecological balance. Among these, typical bisphenol-based EDCs, notably bisphenol A (BPA), are prevalent in industrial applications due to their thermal stability, strength, plasticity, and cost-effective synthesis [1,2]. Despite BPA being regulated or banned worldwide due to its proven reproductive toxic effects, the structurally similar substitutes for bisphenol S (BPS), bisphenol F (BPF), and bisphenol AF (BPAF) have been introduced to produce BPA-free products, such as electronic materials, bottles, food packaging, clothing, and even dental fillings [2]. Noteworthily, recent studies have confirmed that BPS, BPF, and BPAF exhibit similar toxic effects to BPA [3,4,5,6], and these analogs have been constantly detected in various environmental media with observed rising trends [6,7]. BPS has been detected in surface water samples from Japan, South Korea, China, and India, with concentrations reaching up to 7.2 μg/L [8,9,10]. The survey results in 2022 showed that BPA (34.9 ng/L) and BPS (24.8 ng/L) were the main bisphenol pollutants in the surface water of the Pearl River in China, accounting for 68% of the total. Furthermore, among 315 urine samples from the United States and seven Asian countries, BPS was found in 81% of the samples, with an average concentration of 0.654 ng/mL [11]. EDCs can enter aquatic animal bodies through the food chain or direct contact, potentially causing dysfunctions in the endocrine, reproductive, developmental, nervous, and immune systems [12,13], while aquatic animals are particularly susceptible to bisphenol contamination [14]. Notably, these BPA analogs demonstrate greater stability and persistence in aquatic environments and their bioavailability is much higher than that of BPA [15,16]. BPA has been demonstrated to possess endocrine-disrupting effects in aquatic organisms, such as sexual aberration, a sex ratio change, reproductive disorders and developmental abnormalities in male animals, and precocious puberty and tumor development in females [17,18,19]. The comparison of endocrine-disrupting effects and toxic mechanisms on BPA and its substitutes towards aquatic organisms’ reproduction has garnered considerable attention.

Estrogen is widely recognized for its crucial role in sexual maturation and differentiation, primarily through transcriptional activation mediated by ligand-induced estrogen receptors (ERs) [20,21]. Among the molecular targets of BPA, its interaction mechanism with the ER has been confirmed in several biological models. Among them, this study of pregnant rats found that BPA could increase the level of progesterone receptor immunoreactivity significantly in the anterior nucleus of the optic nerve of the female offspring under the activation of Erα [22]. During vivo zebrafish experiments, BPA and BPAF can mediate ER activity and activate transgenic Tg expression at low concentration levels in the heart and higher concentration levels in the liver of young fish, respectively [23]. Additionally, more evidence suggests that ERs also exist in mollusks [23,24,25,26,27]. The correlation between ER mRNA and E2 levels in the ovaries at different stages of maturation of *Crassostrean angulata* and *Chlamys farreri* presents that a similar response to endogenous estrogens may also exist in bivalves [24]. In the study of *Mytilus edulis* and *Mytilus galloprovincialis*, it was found that ER had the ability to automatically regulate its expression in gonadal cells through estrogen, and ER responded to estrogen compounds [25]. The discovery of *Saccostrea glomerata* in oysters has found that ER mRNA expression was highest in the ovaries, and after exposure to 17β-estradiol (E2) in vitro and in vivo, ER mRNA expression in the ovaries was significantly upregulated [26]. The above evidence confirms that ER plays an important role in the reproductive process of mollusk. However, because the ER of invertebrates is significantly different in its structure from that of vertebrates, the mechanism of the binding and activation to EDCs still needs to be further studied.

Delfosse et al. (2012) used a computer homology simulation to explore the interaction of ERα with BPA and BPAF, and confirmed that BPA and BPAF can regulate the transcriptional activity of human ER in Hela cells [27]. As benthic organisms, bivalves can accumulate EDCs through filtration and feeding and make them important indicator organisms to evaluate aquatic pollution. Homology modeling and molecular docking techniques have been used to investigate the stable binding between paralytic shellfish toxin-binding protein of the oyster *Crassostrea hongkongensis* and paralytic shellfish toxin (PST) [28]. Currently, there is no direct evidence that bivalves produce E2 or that their ERs are involved in endocrine regulation [29]. Liu et al. (2021) studied the interactions between ERs and E2, benzopyrene (B[a]P), tetrabromobisphenol A (TBBPA), and 4-Nitrophenol (4-NP) in *C. farreri*, and identified the ER active sites associated with estrogen activity and their binding affinity to ligands for the first time [30]. Thus, homology modeling and molecular docking techniques are effective ways which can predict the binding mode and free energy of small molecules and receptors, providing key molecular evidence for understanding the binding properties of ERs’ active sites and EDCs on bivalves. Pan et al. (2024) employed molecular docking technology combined with drug resistance experiments and qRT-PCR to explore the mechanism underlying the resistance of *Acinetobacter baumannii* to carbapenem antibiotics and found that docking energy values and gene expression levels could mutually validate each other [31]. Additionally, in a study investigating the effects of dichlorodiphenyltrichloroethane (DDT) on the endocrine system and toxicity in killer whales, the authors used molecular docking methods and measured the transactivation potential of DDT, revealing a significant positive correlation between the in vitro transactivation potential values and computer-simulated docking scores [32]. The combination of molecular docking technology and exposure experiments provides a new perspective for exploring the toxic effects of EDCs.

In this experiment, the model organism *Danio rerio* and the ERs of three bivalve species (*Corbicula fluminea*, *Azumapecten farreri*, and *Ruditapes philippinarum*) were selected as the study subjects. The full-length cDNA of *C. fluminea* ER (*Cf*ER) was cloned using the RACE technique for the first time. Homology models of ERs’ LBD from *D. rerio*, *C. fluminea*, *A. farreri*, and *R. philippinarum* were constructed via homology modeling. The best model was screened, and natural estrogen E2, BPA, and its substitutes (BPS, BPF, and BPAF) were docked to this model to explore pollutant affinity and docking sites to ERs in these species. Single exposure experiments were conducted under environmental concentration settings to explore the connection between the docking results and gene expression levels. This study is the first to integrate computer modeling with exposure experiments to characterize the ER toxicity potency and binding sites of BPA and its substitutes in bivalves, and to conduct a reproductive risk assessment of these compounds.

## 2. Results

### 2.1. Bioinformatics Analysis of CfER

#### 2.1.1. Sequence Analysis of *Cf*ER

**Figure 1:**
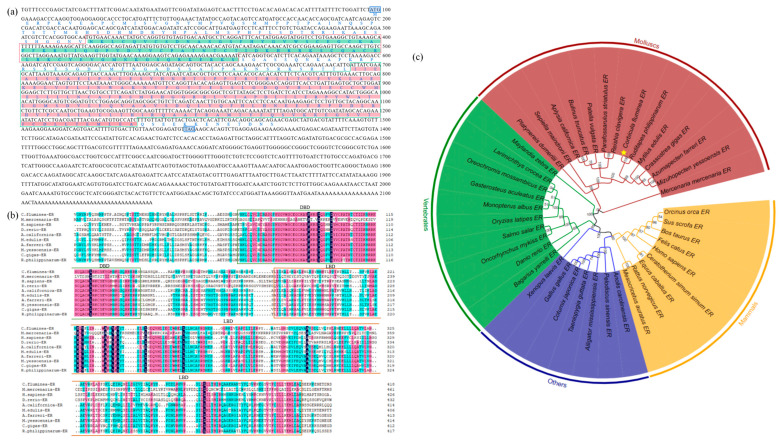
Cloning sequence and bioinformatics analysis of *CfER* genes. (**a**) *Cf*ER cDNA sequence cloned by RACE and its predicted coding amino acid sequence. DBD and LBD sequences are shaded in green and orange, respectively. (**b**) *Cf*ER sequence alignment; DBD and LBD are boxed with green and orange borders, respectively. (**c**) Phylogenetic tree of ER amino acid sequences from multiple species. Branching point numbers indicate bootstrap values. Mollusk, vertebrate, and mammal branches are highlighted with red, green, and blue backgrounds, respectively. GenBank sequences in the tree include *Mercenaria mercenaria* ER: XP_053380899.1, *Mizuhopecten yessoensis* ER: BAJ07192.3, *Azumapecten farreri* ER: FJ516744.1, *Crassostrea gigas* ER: BAF45381.1, *Mytilus edulis* ER: BAF34366.2, *Ruditapes philippinarum* ER: MN542915.1, *Corbicula fluminea* ER: OR365079, *Reishia clavigera* ER: BAC66480.2, *Parafossarulus striatulus* ER: ATU32602.1, *Patella vulgata* ER: XP_050404986.1, *Bulinus truncatus* ER: KAH9509098.1, *Aplysia californica* ER: AAQ95045.1, *Sepiella maindroni* ER: AMR55387.1, *Platynereis dumerilii* ER: ACC94156.1, *Maylandia zebra* ER: XP_004554745.1, *Larimichthys crocea* ER: NP_001290305.1, *Oreochromis mossambicus* ER: CAK95869.1, *Gasterosteus aculeatus* ER: NP_001254601.1, *Monopterus albus* ER: AAT95432.1, *Oryzias latipes* ER: BAA86925.1, *Salmo salar* ER: NP_001117064.1, *Oncorhynchus mykiss* ER: NP_001117821.1, *Danio rerio* ER: AB037185.1, *Bagarius yarrell*i ER: TSR99413.1, *Xenopus laevis* ER: NP_001083086.1, *Gallus gallus* ER: NP_990514.1, *Coturnix japonica* ER: NP_001310118.1, *Taeniopygia guttata* ER: NP_001070169.1, *Alligator mississippiensis* ER: NP_001274203.1, *Pelodiscus sinensis* ER: WFD51103.1, *Anolis carolinensis* ER: NP_001277446.1, *Mesocricetus auratus* ER: NP_001268254.1, *Rattus norvegicus* ER: NP_036821.1, *Equus caballus* ER: NP_001075241.1, *Ceratotherium simum simum* ER: NP_001266182.1, *Homo sapiens* ER: AAA52399.1, *Felis catus* ER: NP_001019402.1, *Bos taurus* ER: NP_001001443.1, *Sus scrofa* ER: NP_999385.1, and *Orcinus orca* ER: XP_033260665.1. The target gene *Cf*ER was marked with a yellow five-pointed star.

The nucleotide sequence of the *Cf*ER gene was successfully cloned and submitted to NCBI (OR365079), revealing a core sequence of 2138 bp, consisting of an 843 bp 3′ non-coding region and a 1404 bp open reading frame (ORF). The ORF encoded 432 amino acids. The computational analysis using ProtParam (http://web.expasy.org/protparam/) (accessed on 10 March 2024) online website indicates that the theoretical molecular weight of *Cf*ER is 4.85 kDa, with an isoelectric point of 7.58. Additionally, the aliphatic amino acid index is 78.36 and the average hydropathicity coefficient is −0.435. The nucleic acid sequence and predicted amino acid sequence of *Cf*ER are illustrated in Figure 1a.

#### 2.1.2. Homology Analysis of *Cf*ER

To explore the similarities between *C. fluminea* and other species, this study compared the full amino acid sequence and LBD-binding domain of ER of *C. fluminea* with those of *M. mercenaria*, *H. sapiens*, *D. rerio*, *A. californica*, *M. edulis*, *A. farreri*, *M. yessoensis*, *C. gigas*, and *R. philippinarum*. As shown in Figure 1b, the binding domains of the DNA-binding domain (DBD) and LBD sequence exhibit a high degree of similarity. The amino acid sequence identity of ER between the species also varies among different domains. As illustrated, the sequences and LBD domains of several species exhibit a high degree of similarity (Figure 1b). Among them, *A. farreri* and *R. philippinarum* show higher similarity to the nucleic acid sequence of *C. fluminea*, with a higher LBD sequence homology.

Phylogenetic analysis revealed four major clades of ERs: mollusks, vertebrates, mammals, and other animals (amphibians and avians). These three types of ERs mentioned above may represent the evolutionary process of animal estrogens. Figure 1c reveals the four main branches of ER in mollusks, vertebrates, mammals, and other animals (amphibians and birds), and the above three types of ERs could represent evolutionary divergence. By combining multiple sequence alignments, it can be observed that the amino acid sequence identity of ER among different species also varies due to different structural domains. The similarity of LBD between *Cf*ER and selected mollusks is 58.8–66.5% and the similarity between *Cf*ER and other vertebrates is lower (35.5–36.4%).

Combined with the genetic distance and phylogenetic analysis, it was found that *A. farreri* and *R. philippinarum* are highly closely related to *C. fluminea*. In toxicological studies, *C. fluminea* is used as an indicator organism in freshwater, while *A. farreri* and *R. philippinarum* are often employed as indicator organisms in marine ecotoxicological research [33,34]. Therefore, *A. farreri* and *R. philippinarum*, together with *C. fluminea*, were selected as research subjects to study BPA and its substitutes in the aquatic environment.

#### 2.1.3. Secondary Structure Prediction of *Cf*ER

**Figure 2:**
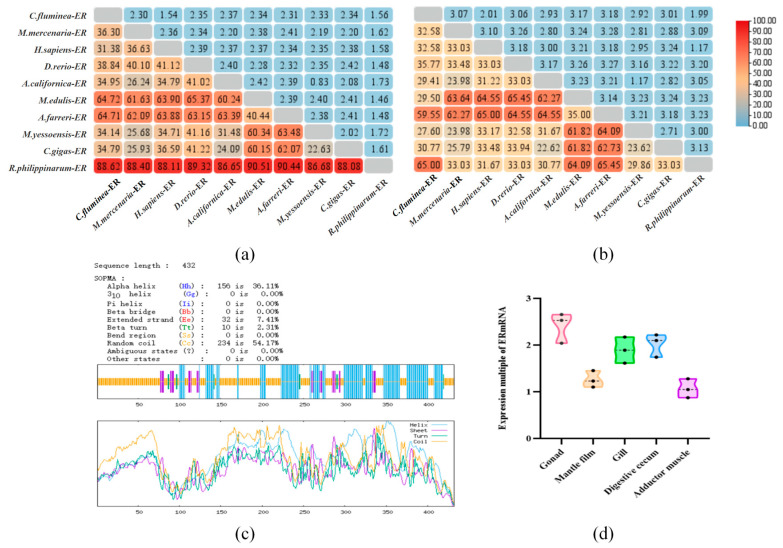
The genetic distance from genes of other species, secondary structure, and tissue-specific expression of *Cf*ER sequences. (**a**) Genetic distance of the full-length *Cf*ER sequence from genes of other species. (**b**) Genetic distance of the *Cf*ER-LBD sequence from genes of other species. Color intensity (redder hues) indicates higher similarity. (**c**) Secondary structure prediction of the *Cf*ER gene sequence. (**d**) Expression of ER in various tissues of *C. fluminea*.

SOPMA (https://npsa-prabi.ibcp.fr/NPSA/npsa_sopma.html) (accessed on 15 April 2024) analysis of the *Cf*ER amino acid sequence shows that its secondary structure consists of α-helix 42.82%, extended strand 8.33%, β-turn 4.17%, and random coil 44.68%, as shown in in Figure 2c. Analysis using the software PSORT II Prediction (https://www.genscript.com/tools/psort) (accessed on 16 April 2024) found that *Cf*ER belongs to a nuclear protein 91.3% and a nuclear hormone receptor’s DNA-binding region signature.

#### 2.1.4. The Situation of Tissue-Specific Expression of *Cf*ER

The expression of the *Cf*ER gene was measured in five tissues (Figure 2d) and the *Cf*ER mRNA had the highest expression in the gonad which implied that it has an important role in reproduction.

### 2.2. Computer Homology Modeling of ERs with BPA and Its Substitutes

#### 2.2.1. Preparation of Small Molecular Ligands

**Figure 3:**
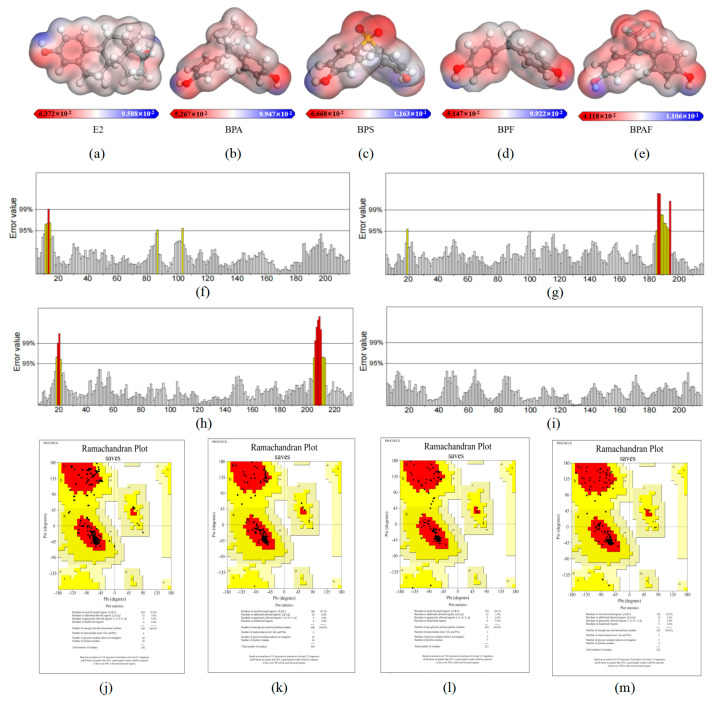
Electrostatic potential (ESP) maps of E2 and BPA and its substitutes, and evaluation results of ERRAT and PROCHECK models for homology models of LBD by *D. rerio*, *C. fluminea*, *A. farreri*, and *R. philippinarum* ERs. (**a**–**e**) represent the ESP diagrams of E2, BPA, BPS, BPF, and BPAF in sequence. The ESP value is represented on a color gradient bar, with red representing negative charge, blue representing positive charge, and white representing neutral charge. (**f**–**i**) are ERRAT evaluation graphs of ERsLBD three-dimensional structures of *D. rerio*, *C. fluminea*, *A. farreri*, and *R. philippinarum*, in turn. (**j**–**m**) are the Lagrange conformation maps of *D. rerio*, *C. fluminea*, *A. farreri*, and *R. philippinarum* ERs LBD, respectively, which are divided into four regions, namely, the red core region (the most favorable region), the yellow generally reasonable region (additional allowable region), and the red core region. Light yellow acceptable zone (generous allowed zone) and white unreasonable zone (not allowed zone).

The electrostatic potential (ESP) of a compound represents the electrostatic potential energy carried by charged particles (such as electrons or protons) at any point in the space surrounding the molecule, which is influenced by the charged components of the molecule [35]. This descriptor is crucial for understanding intermolecular interactions, particularly the interactions between the hydrophobicity of the ER and the molecules of BPA and its substitutes. In Figure 3a–e, the blue areas represent the most positive potential, which is responsible for nucleophilic interactions, while the red areas indicate the most negative potential, representing the sites of electrostatic interactions.

The analysis of the electrostatic potential energy of the pollutants revealed that, like the hydroxyl group of E2, the structures of the four pollutants all consist of two benzene rings linked to hydroxyl groups. The regions with strongly negative electrostatic potential are concentrated on the hydroxyl groups attached to the benzene rings (negative charge regions are shown in red in Figure 3a–e). Due to their electronegativity, these regions can significantly affect molecular interactions, thereby increasing the binding affinity of amino acid residues in the LBD of ERs.

#### 2.2.2. Homologous Modeling of ERs from *D. rerio* and the Three Bivalves

Due to the scarcity of crystal diffraction structures for fish and shellfish, studies on the molecular docking of shellfish often use the human ER crystal structure as a template to construct homologous models of shellfish [36]. In this study, homologous models of the ERs LBD for *D. rerio*, *C. fluminea*, *A. farreri*, and *R. philippinarum* were constructed using the human ER crystal structure as a template. It is generally accepted that when the sequence homology of the target gene’s LBD with the template sequence exceeds 50%, the accuracy of the predicted three-dimensional structure of the target protein is very high. If the sequence homology ranges from 30% to 50%, the precision of the three-dimensional structure is within an acceptable range, whereas a homology below 30% results in relatively low structural precision.

Human ER crystal templates with over 30% homology to the target genes were selected for constructing the ER LBD protein sequences of *D. rerio*, *C. fluminea*, *A. farreri*, and *R. philippinarum*, as summarized in Table 1. These templates were further screened by comparison with the modeller database and protein data bank (PDB). Through comprehensive searching, a series of protein X-ray diffraction crystal structures with high homology to the target genes were obtained.

QMEAN evaluates structural quality based on physicochemical properties, provides an overall quality score, and compares it with the estimated QMEAN scores of high-resolution experimental structures. A QMEAN score close to 0 indicates a good agreement between the homology model and the true structure. Among the constructed models, the one with the highest absolute DOPE score was selected, as this indicates a more accurate conformational template. The constructed three-dimensional structures of ER-LBD from *D. rerio*, *C. fluminea*, *A. farreri*, and *R. philippinarum* contain 238, 221, 221, and 221 amino acid residues, respectively.

#### 2.2.3. Evaluation and Analysis of ERs Model from *D. rerio* and the Three Bivalves

Then ERRAT and PROCHECK were used to evaluate the reliability of the homology model. Table 2 shows the comprehensive results of the quality check parameters of the three models. The ERRAT model evaluation method mainly evaluates the quality of the 3D model by evaluating the random distribution of different types of atoms in the model. The error axis of ERRAT generally has two lines representing the confidence that the rejectable region exceeds the error value in Figure 3f–i. The overall quality value is expressed as a percentage and the average overall quality factor is generally 90%. In this study, the ERRAT values of the ERs LBD three-dimensional structure were 94.6%, 100.0%, 97.2%, and 94.8% for *D. rerio*, *C. fluminea*, *A. farreri*, and *R. philippinarum*, respectively. The ERRAT values of the four models are all greater than 90%, indicating that they exhibited high-resolution structures and can be considered to have a reasonable atomic distribution.

In Figure 3j–m, in the PROCHECK model evaluation, the Lagrange conformation map is categorized into four areas, namely, the red core area (the most favorable area), the generally reasonable yellow area (the additional allowable area), the light-yellow acceptable area (the generous allowable area), and the white unreasonable area (the disallowed area). Each black data point in the figure corresponds to the amino acid residue of the model, representing the correspondence between the two dihedral angles of the amino acid residue at the Ca atom. A high-quality model, in theory, should have over 90% of its amino acid residues located in the core region. The statistical analysis results showed that the proportion of amino acid residues in the core region of the *D. rerio*, *C. fluminea*, *A. farreri*, and *R. philippinarum* LBD three-dimensional structure was 92.4%, 95.1%, 94.1%, and 93.7%, respectively, and the results were all greater than 90%. It is concluded that the four models constructed in this study have good stereochemical characteristics and are reasonable models of high quality.

Considering the docking score and model structure, the selected optimal docking conformation and corresponding energy are shown in Table 3. Among them, the active center (center grid box) of *D. rerio* ER LBD is at x = 23.277, y = 39.483, and z = −88.054 (with a spacing of 0.553); the active center of *C. fluminea* ER LBD is at x = −0.577, y = −0.574, and z = −34.224 (with a spacing of 0.536); the active center of *A. farreri* ER LBD is at x = 9.028, y = −4.521, and z = −0.667 (with a spacing of 0.458); and the active center of *R. philippinarum* ER LBD is at x = −24.648, y = −1.348, and z = 22.202 (with a spacing of 0.542). The binding free energy is calculated by adding the final intermolecular energy, total internal energy, and torsion-free energy, then subtracting the energy of the unbound system; the docking energy is the sum of intermolecular energy and intramolecular energy.

**Figure 4:**
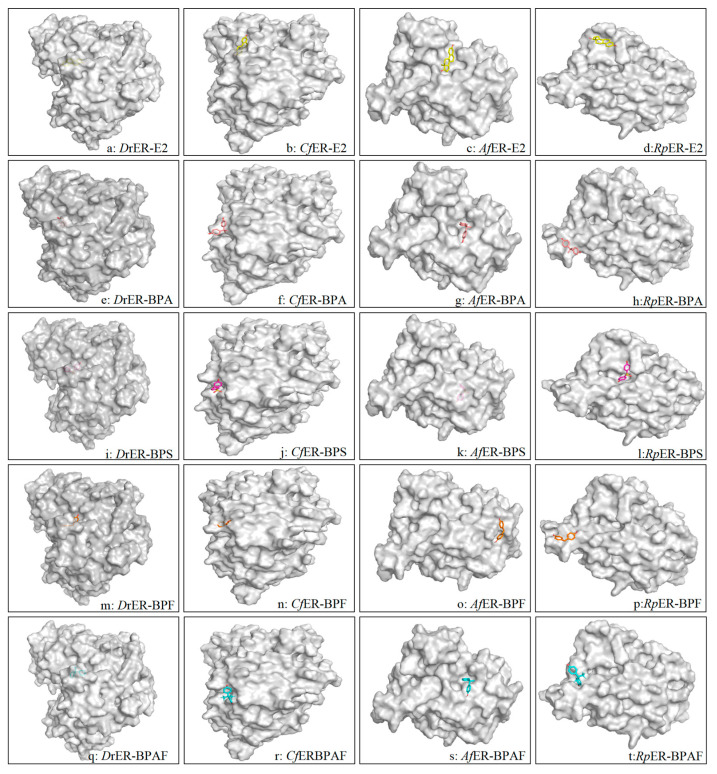
*D. rerio*, *C. fluminea*, *A. farreri*, and *R.philippinarum* are surface structure model diagrams formed by the connection with E2, BPA, BPS, BPF, and BPAF, respectively. Species–pollutant combinations labeled in the lower right corner. Gray regions depict protein structures of different substances, with central 3D stick models representing pollutants: E2 (yellow), BPA (light pink), BPS (purple), BPF (orange), and BPAF (blue).

The interactions between the estrogen receptors of zebrafish and several bivalve species with various bisphenol pollutants were predicted using Autodock4 software. The autodock result files contain several parameters for ranking docking conformations, with three key pieces of information primarily utilized: (i) the affinity between the ligand and the receptor binding pocket and/or the stability of the ligand–receptor complex, (ii) the interactions between ligand atoms and target amino acid residues, and (iii) the distances of these interactions. The docking results revealed consistent binding modes formed between several bisphenol pollutants and three types of bivalves and *D. rerio*. The three-dimensional docking models demonstrated hydrophobic interaction pockets between the pollutant ligands and binding site residues. The ligands are displayed as ball-and-stick models, and the residues in contact with the ligand molecules are shown in gray, as illustrated in Figure 4.

### 2.3. Protein–Ligand Docking Analysis

#### 2.3.1. Docking Conformation Analysis of ERs and E2

**Figure 5:**
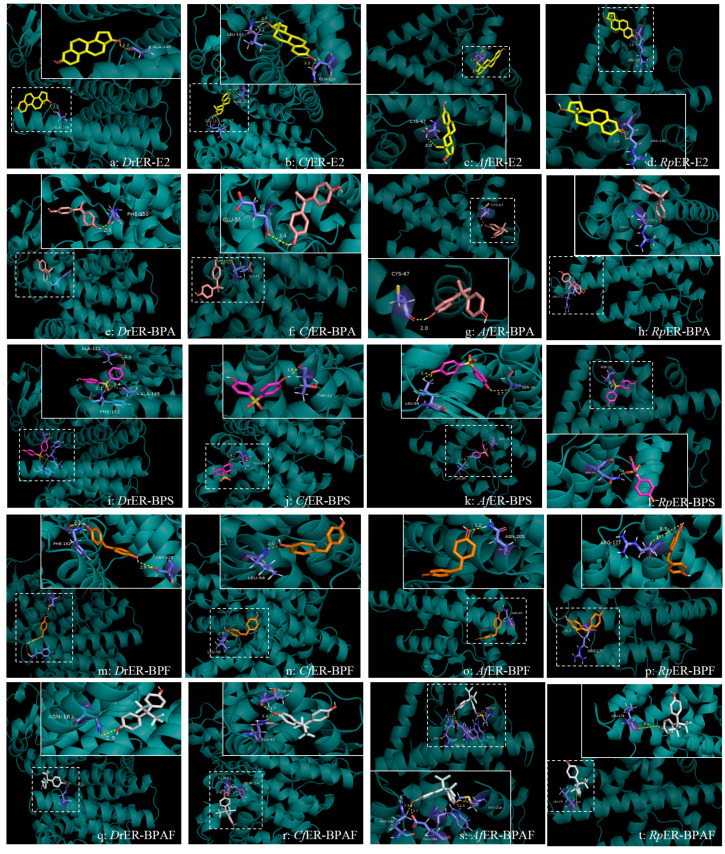
Prediction analysis diagram of interaction between amino acid residues and various pollutants of *D. rerio*, *C. fluminea*, *A. farreri*, and *R. philippinarum*. Row: species–pollutant interaction results; column: same pollutant across different species. Each figure consists of a whole picture and a partial picture. The white dashed box encircles the binding sites of the pollutants on the macromolecular proteins, and a local diagram with a white solid box is presented beside it to show the specific binding situation. The species–pollutant label is located at the lower right corner. Macromolecular protein 3D structures in cyan; pollutants as stick models (E2: yellow, BPA: pink, BPS: purple, BPF: orange, and BPAF: grey).

The docking results of ERs with E2 in *D. rerio*, *C. fluminea*, *A. farreri*, and *R. philippinarum* are shown in Figure 5a–d. As depicted, the hydroxyl groups on the benzene ring of E2 can bind to two amino acid residues (Leu-147 and Gln-123) of *Cf*ER, forming three hydrogen bonds, whereas only one hydrogen bond is formed in the other three species. Furthermore, according to the energies generated during docking (as shown in Table 3), the binding energy and docking energy of *Cf*ER with E2 are −5.54 kcal/mol and −12.22 kcal/mol, respectively. Compared with the other three species, *Cf*ER releases more energy during docking and thus binds more easily. For *Rp*ER, the binding energy and docking energy with E2 are −3.52 kcal/mol and −8.13 kcal/mol, respectively, showing the least energy release during docking and lower affinity with E2 compared to the other three species. Based on the binding energy, docking energy, and other reference energies, the order of binding affinity of the ER LBD homology models of the four species with E2 is *C. fluminea* > *D. rerio* > *A. farreri* > *R. philippinarum*.

#### 2.3.2. Docking Conformation Analysis of ERs and BPA

Regarding the binding process between ERs and BPA, as can be seen from Figure 5e–h, the ERs of all four species can bind to BPA, with each forming only one hydrogen bond. The hydrogen bond formed between *Cf*ER and BPA has a longer distance of 3.4Å, and a shorter hydrogen bond distance indicates relatively greater stability. The hydrogen bonds formed by the docking of *Dr*ER and *Af*ER with BPA are both 2Å, showing relatively higher stability. Referring to the docking energies in Table 3, *Rp*ER exhibits the smallest absolute values of binding energy and docking energy, indicating that *Rp*ER has the lowest affinity for BPA compared to the other three species. With consistent torsional energy, the order of the binding affinity to BPA, based on a comprehensive consideration of docking energy, binding energy, and ligand efficiency, is *D. rerio* > *A. farreri* > *C. fluminea* > *R. philippinarum*.

#### 2.3.3. Docking Conformation Analysis of ERs and BPS

In the docking analysis of ERs with BPS, it was found that the docking energy of *Af*ER with BPS was the lowest at −4.23 kcal/mol, but only two hydrogen bonds were formed. Although the docking energy of *Dr*ER was higher than that of *Af*ER, *Dr*ER could form three hydrogen bonds with BPS through docking, indicating that *Dr*ER had the highest affinity for BPS. Both *Cf*ER and *Rp*ER formed only one hydrogen bond with BPS during docking, and the hydrogen bond distances were the same at 1.9Å. However, the docking energy of *Cf*ER with BPS was higher than that of *Rp*ER. With reference to values such as the ligand efficiency and binding free energy, the order of affinity of the four species for BPS was as follows: *D. rerio* was the highest, followed by *A. farreri* and *R. philippinarum*, with *C. fluminea* being the lowest.

#### 2.3.4. Docking Conformation Analysis of ERs and BPF

In the docking analysis of ERs and BPF, shown in Figure 5m–p, it was found that *Dr*ER has two amino acid residues that dock with BPF, forming two hydrogen bonds (Phe-152, Ser-128). The three-dimensional structural models of the other three bivalves only have one amino acid residue that binds to BPF and only forms one hydrogen bond. The hydrogen bond between amino acid residues of *Cf*ER and BPF is the shortest and more stable. According to the binding energy and length of each hydrogen bond formed, and with reference to the ligand efficiency, the order of affinity of each species for BPF was finally obtained as follows: *D. rerio* > *C. fluminea* > *A. farreri* > *R. philippinarum*.

#### 2.3.5. Docking Conformation Analysis of ERs and BPAF

In the docking analysis of ERs and BPAF (Figure 5q–t), it was found that the four amino acid (Arg-190, Glu-194, Trp-69, and Met-218) residues of *Af*ER can dock with BPAF to form four hydrogen bonds, and the two amino acid residues of *Cf*ER can dock with BPAF to form two hydrogen bonds. According to Table 3, the binding energy of *Af*ER is greater than *Cf*ER. Referring to the binding free energy, the order of affinity between several species and BPAF was finally obtained: *A. farreri* > *C. fluminea* > *D. rerio* > *R. philippinarum*.

### 2.4. RT-qPCR Analysis of ERs Expression Under Exposure of E2 and Selected Typical Bisphenol EDCs

**Figure 6:**
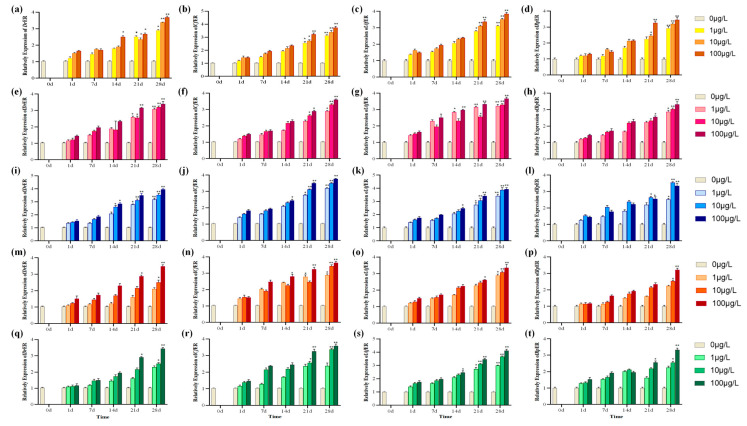
The effect of ER gene expression in gonadal tissues of various species under single stress of E2 and BPA and their substitutes within 28 days of exposure. (**a**–**d**) ER gene expression in gonads of four species under E2 stress; (**e**–**h**) under BPA; (**i**–**l**) under BPS; (**m**–**p**) under BPF; and (**q**–**t**) under BPAF. Asterisks denote statistical significance vs. control (* *p* < 0.05; ** *p* < 0.01).

To assess and verify whether the toxic effects of the above EDCs on ER genes of tested species are consistent with the molecular docking results, additional exposure experiments were conducted (Figure 6). The pre-experiments had indicated that there was no significant difference between the DMSO control group and the blank control group. The melting curve has a clear outline and a single peak. Before the 7 days’ exposure experiment, no significant difference was observed in the ER mRNA levels between the exposure groups and the control group (*p* > 0.05). It was found that at the later stage of the experiment, with the increase in the concentration of EDCs, the expression levels of ER mRNA gradually increased and the ER mRNA expressions increased significantly at 100 μg/L (*p* < 0.001).

As shown in Figure 6, under the stress exposure of BPA and its substitutes, it can be observed that the expression levels of ER mRNA in all four species showed a significant increase in the later stage of the experiment (*p* < 0.05). Among them, under the exposure of E2 and BPA, it could be observed that the expression level of the ER mRNA of *A. farreri* increased extremely significantly in the later stage of the experiment (*p* < 0.001) and it was more sensitive to BPA compared with the other three species. Under BPS exposure, as shown in Figure 6i–l, it can be observed that the ER mRNA expression levels of *A. farreri* and *C. fluminea* increased significantly compared to other species, and all three concentrations showed extremely significant increases in the later stage of the experiment (*p* < 0.001). In the later stage of the experiment, although *R. philippinarum* also showed a significant increase (*p* < 0.05), it was less sensitive to BPS compared with the other three species. Under BPF exposure, *C. fluminea* showed greater sensitivity. At 28 days, at concentrations of 10 μg/L and 100 μg/L, it exhibited extremely significantly increased phenomena (*p* < 0.001). Under BPAF exposure, the expression levels of ER mRNA in *A. farreri* and *C. fluminea* increased relatively more significantly, and both showed higher affinity for BPS, BPF, and BPAF.

## 3. Discussion

### 3.1. The Evolutionary Analysis of ERs in Mollusks

At present, only ER, the most primitive steroid hormone receptor, has been found in the genome of mollusks, and the original ER has been confirmed to be the origin of two (ERα and ERβ), the androgen receptor and progesterone receptor in vertebrates [28], whereas only one of the ERs’ subtypes has been found in mollusks [37]. The ER homologous sequence has been cloned in many species of mollusks, such as *C. farreri*, *C. angulata*, *C. gigas*, *M. edulis*, and *M. galloprovincialis*, etc., [24,25,37,38], which have been confirmed to be related to the reproductive function. This study selected one typical freshwater bivalve, *C*. *fluminea*, and cloned the full-length cDNA sequence of ER, finding that the expression of the *Cf*ER gene was highest in the gonad, which suggested the reproductive role of ER.

However, in terms of the molecular evolution, it is generally believed that steroid receptors (SRs), including ERs, originate from a single ancestral receptor [39,40,41]. In a phylogenetic context, the SRs family consists of two subgroups, one of which is the ER, and other subgroups are the mineralocorticoids receptor (MR), glucocorticoids receptor (GR), and androgens receptor (AR). AR and progestogen receptors (PRs) are included in the latter subgroup due to the clear homology of the mollusk ERs sequence to SRs and its closest similarity to vertebrate ERs [39,42]. The researchers analyzed three subtypes of vertebrates (mammal, bird, reptilia, amphibian, bony fish, and elasmobranch). Phylogenetic relationships exist between invertebrates (annelidas and mollusks) and cephalochordate ERs [43]. Consistent with previous studies, the results in this study support the hypothesis that ancestral receptors evolved into two sister clades of vertebrate ERs and invertebrate ERs, with mollusks and annelids being grouped into the latter clade and performing similar functions. From the evolutionary tree, *Cf*ER is closely related to the invertebrate mollusks’ ERs, which further implies that *Cf*ER and vertebrate ERs are different in their structure and function.

In addition, *Cf*ER-derived protein sequences of the DBD domain and LBD domain were highly conserved by multiple comparison analysis with the ERs gene of other mollusks and ERα gene of vertebrates. The LBD domain includes ligand-dependent transcriptional activation associated with ligand linking, dimerization, and binding, and the results revealed that this sequence may interact with estrogen [44]. In the present study, *C. fluminea* shows a high degree of similarity (up to 65%) with *A. farreri* and *R. philippinarum*. However, the LBD sequence of *C. fluminea* ER shares a low similarity (only 30%) with that of vertebrates (humans and zebrafish). It is inferred that this may be due to the distant phylogenetic relationship between invertebrates and vertebrates, resulting in significant interspecific differences.

### 3.2. The Application of Homologous Modeling and Molecular Docking in Toxicology

More than 80,000 chemicals have been released into the human environment over the past century. The Organization for Economic Co-operation and Development (OECD) outlines various experimental methods for assessing estrogenic active chemicals, such as reporter gene-based assays, receptor binding and dimerization assays, and cell proliferation assays [45,46]. The number of these unknown estrogenic active chemicals far exceeds the number of chemicals that can be tested using various systems, and an effective toxicological evaluation of all chemicals is difficult to complete. At present, homology modeling and molecular docking have become essential components of numerous computational simulations based on chemical structures [47,48]. Homology modeling is a method that uses determined X-ray crystallographic data to predict the conformation of another protein with a similar amino acid sequence [49]. Many studies have confirmed that some EDCs could affect ER signaling directly by competing with endogenous steroids for binding sites on the ER, which include pesticides of thiosulfan, tri-butylstannane (TBT), and industrial raw materials and products of PCBs, diphenylacetone, and phenols of NP, TBBPA, DDT, and other substances like dioxin, B[a]P, etc., [30,50,51].

Molecular docking has been widely used to observe the interaction between ligands and receptors. For example, Celik et al. (2008) found that PCBs, plasticizers, and pesticides can all bind to human ER, and the receptor interacts with at least one of the two hydrophilic ends of these steroid-binding sites [42,52]. Tohyama et al. (2015) reported that the electrostatic interaction between amino acid residues and hydroxyl groups at C3 and C17 is crucial for the activation ability of ERs, which represents that a distinct feature of ER is their ability to bind estrogen with high affinity [53]. Previous reports showed that hydrogen bonds between human ER and E2 are formed by amino acid residues Glu-353, Arg-394, and His-524. The main amino acid residues that form hydrogen bonds between ER and the E2 ligand in fish are the same as those in human ER, namely Glu-321 (Glu-353), Arg-362 (Arg-394), and His-492 (His-524). During the docking process in this study, the amino acid residues formed in the five homologous model ligand-binding pockets were highly conserved. We noted that the active site of Glu-353 in the human eutectic model corresponds to *Oryzias latipes* (ER2α: Glu-316, ER2β: Glu-355) and rotifers (Glu-9) [53], and also corresponds to the docking sites of *A. farreri* (Glu-66, Glu-55, and Glu-194) in this study. The main amino acid residue that *R. philippinarum* binds to is Arg-177, which is equally similar to that of humans and fish. This indicates that the amino acid residue may have a key function during the binding process with the ligand. Studies have revealed that several key residues, including Glu-305, Met-336, Leu-339, Met-340, Leu-343, Arg-346, Met-473, and Leu-476, play crucial roles in the binding process of human ERs with ligands [54]. In the present study, the amino acid residues that play key roles in the binding of ERs with BPA and its alternatives in three bivalve species include *C. fluminea* (Leu-147, Leu-58), *A. farreri* (Leu-89, Met-218), and *R. philippinarum* (Leu-184, Leu-176), which show similarities to relevant studies on human ERs. However, other functional amino acid residues identified in this study, such as Ala-49, Ala-121, Phe-152, and Cys-67, differ from those reported in previous studies on vertebrates. This discrepancy may be attributed to differences in the active site structures of ER proteins between mollusks and vertebrates, leading to variations in the mechanism of action of ERs. Additionally, during the docking process, it was observed that different pollutants share similar docking sites when binding to ERs. For instance, *D. rerio* can bind to the Ala-149 site with both E2 and BPS, *A. farreri* can bind to the Cys-87 site with both E2 and BPA, and *R. philippinarum* can bind to the Arg-177 site with BPA and BPF. These findings indicate that different pollutants may share common binding sites with ligands, potentially resulting in similar toxicological mechanisms. Our laboratory will conduct in vitro experiments to further explore the mechanism of action of mollusks in the future.

Generally, the lower the free binding energy, the more stable the ligand–receptor binding is in general [55,56]. According to the binding energy of the docking results in this study, it was found that *A. farreri* combined with BPS had the lowest binding energy, indicating that the combination of the two was stable, while *D. rerio* combined with BPAF had the highest binding energy, indicating that the two were unstable, as shown in Table 3. However, the number of unknown estrogen-active chemicals is much larger than the number of chemicals that can be tested based on various systems, and it is difficult to perform an effective toxicological assessment of all chemicals [57]. This experiment lays a foundation for exploring the structure and function of ER and the mechanisms of the endocrine disruption of EDCs in bivalves. It also provides a theoretical basis and technical support for the toxicological monitoring of the water environment. However, it is inevitable that docking results can only theoretically predict the interaction sites and affinity between different pollutants and ligands, and there must be certain discrepancies from the actual binding situations. The toxic effects under in vitro exposure still need to be combined with practical production conditions.

### 3.3. Effects of BPA and Its Substitutes on ER Expression in Mollusks

Studies have shown that EDCs such as B[a]P, BPA, and diethylstilbestrol (synthetic nonsteroidal estrogen) have estrogenic activity and can activate target genes downstream of ER to produce endocrine-disrupting effects [58,59]. In recent years, significant progress has been made in the study of the effects of bisphenol pollutants on ER expression in vertebrates and mollusks [26,60,61]. Tohyama et al. (2015) compared and found that the binding activity of ERα with BPA after the recombinant expression of five different fish was 0.017 to 0.1 times that of E2, which confirmed that BPA had weak estrogen activity [53]. Qiu et al. (2020) found that exposure to 1 ug/L and 100 ug/L of BPA, BPS, BPF, and BPAF would inhibit the growth of zebrafish larvae, cause motor disorders, and promote the gene expression of ERα and ERß [61]. Park et al. (2022) found that 200 μg/mL BPS could induce the significant up-regulation of ER and Vtg genes in female zebrafish [62]. Currently, although mollusks and other aquatic invertebrates account for more than 95% of aquatic biodiversity, they are significantly under-represented in the routine toxicity testing and risk assessment of EDCs [63,64]. It is generally believed that ER plays different biological functions in shellfish than in vertebrates. Mollusks (such as mussels, snails, etc.) are highly sensitive to environmental pollutants, so they become a good indicator species for studying the effects of bisphenol pollutants, and their role in environmental monitoring is gradually being recognized [50,64]. In vivo experiments on two kinds of mollusks, *A. californica* and *O. vulgaris*, found that ER has binding activity with E2, estrone, and other estrogens, and E2 at 10 pM could induce ER gene expression in *O. vulgaris* to be significantly up-regulated [41,65]. Yang et al. (2020) exposed female *C. farreri* to B[a]P for 7 days and found that the P, T, and E2 contents in *C. farreri* were significantly reduced and the expression of the ER gene was decreased [66]. After exposure to TBBPA, the expression of ER is enhanced in muscles, which may lead to the dysregulation of metabolic levels and a decreased reproductive capacity [50]. From the above studies, it can be concluded that the ER of invertebrates is the binding site of estradiol and EDCs with an estrogen effect, and the ER of mollusks may have similar functions to that of vertebrates. In this experiment, as shown in Figure 6, it was found that the ER expression levels of *D. rerio* and three bivalves began to show a significant upward trend after 14 days of exposure to the same pollutant (*p* < 0.05). In this experiment, three experimental concentrations of 1, 10, and 100 ug/L were designed based on the environmental concentration, with a single continuous exposure of 28 days. The results in this study indicate that the ERs’ mRNA expressions were regulated by BPA and its substitutes as well, further confirming the estrogenic effects of these EDCs.

Conclusions about the toxicity of BPA and its analogues in living organisms are also mixed. Rochester and Bolden (2015) reviewed the adverse effects of BPS and BPF on reproduction and determined that these BPA aniloxes have slightly less hormonal efficacy than BPA, but have similar effects on the reproductive system as BPA [67,68]. Mu et al. (2018) found that among BPAF, BPA, BPF, and BPS, the binding potential for zebrafish ER was in the order of BPAF > BPA > BPF > BPS, among which BPAF had the highest estrogen activity and toxicity to zebrafish, and its binding ability to ER was much higher than that of BPA [69]. Like BPA, BPA analogue (BPB, BPF, and BPS) can induce oxidative stress and damage the reproductive system of rats, thereby affecting the ovaries [70]. The conclusions of these studies on BPA and its alternatives have some similarities and differences with the results of this study. However, previous studies were limited to infer the size of toxic effects of several species by measuring ER-related hormone levels and enzyme activity. Suma et al. (2020) utilized molecular docking and spectroscopic experiments to jointly investigate the herbicidal activity of the herbicide atrazine [71]. It has been confirmed that molecular docking can be combined with experiments to jointly explore the toxic effects of pollutants on living organisms [31,32]. Similar to this study, the exposure experiment is combined with the molecular docking technology, and the docking energy given by the computer and the number and length of hydrogen bonds generated by docking were used to comprehensively conclude that the toxicity of BPA and its substitutes was BPA > BPS > BPF > BPAF; the docking results and exposure experiments of ER mRNA could basically verify each other and were more persuasive.

## 4. Materials and Methods

### 4.1. Pollutant Preparation

BPA (Purity ≥ 99%), BPS (≥99%), BPF (≥98.0%), BPAF (≥98.0%), and natural estrogen E2 (≥97.0%) were separately dissolved in DMSO (99.5%, Sigma, Shanghai, China); the DMSO concentration in experimental water environment ≤ 0.005% (*v*/*v*) was safe [72] and diluted in deionized water into mother liquor. The chemical formulas and structures of several pollutants and organic solvents are shown in Appendix A Table A1. To prevent photodegradation, a new bottle of contaminant stock solution was prepared every 7 days and stored in a brown reagent bottle.

### 4.2. Laboratory Animal Culture

Experimental zebrafish were purchased from an experimental zebrafish base in Wuhan, China. Female adult zebrafish of the AB strain were selected and fed 2% of their body weight with special feed in the morning and evening. The 1-year-old *C. fluminea* tested were grown in the freshwater culture system of water environmental toxicology laboratory, College of Animal Science and Technology, Henan University of Science and Technology. The adult *A. farreri* and *R. philippinarum* were taken from the Huanghai Sea seafood market in Qingdao, China, with shell lengths of 7.25 ± 0.5 cm and 2.71 ± 0.46 cm, respectively. The bivalves were fed with chlorella powder and dried spirulina powder (3 g/m^3^) every day. Aquaculture water quality parameters are shown in Appendix A Table A2. After all the experimental animals were temporarily kept for 10 days and their conditions were stable, no bisphenol residues were detected in their bodies after the 10-day acclimation.

### 4.3. RACE Cloning and Tissue-Specific Expression of CfER Gene

#### 4.3.1. RNA Extraction and First-Strand Synthesis of cDNA

Total RNA was extracted from tissue samples using Trizol reagent (Invitrogen Company, Waltham, MA, USA). RNA concentration and purity were determined using nucleic acid concentration meter (Nanodrop, Thermo Scientific, Shanghai, China), using oligo (dT) as primers. cDNA was synthesized by SMARTScribe reverse transcriptase. In this process, the SMARTer II oligonucleotide provides a specific sequence to enable cDNA to have a universal primer-binding site for subsequent PCR amplification.

#### 4.3.2. 5′ RACE Experiment and 3′ RACE Experiment

Primer Premier 5.0 was used to design specific primers GSP1-GSP3. The primers’ sequences are shown in Appendix A Table A3. The first strand of cDNA was synthesized using SUPERSCRIPT II RT enzyme and GSP1 primer, and the RNA was removed. The GLASSMAX DNA purification system was used to remove the residue and ensure the quality of subsequent amplification. A dC tail was added to the 3′ end of cDNA using TdT enzyme to improve PCR amplification efficiency, and the first round of PCR amplification was performed with GSP2 and anchor primer AAP. PCR conditions: pre-transformation at 94 °C for 2 min, 35 cycles at 94 °C for 30 s, 55 °C for 30 s, 72 °C for 1 min, 72 °C for 10 min, and then nested PCR was carried out using the first round of PCR products as the template to improve the specificity. After electrophoretic detection of PCR products, the target fragments were recovered and purified. The purified PCR products were connected to pMD18T vector, transformed into receptor cells, screened for positive clones, and sequenced.

According to the known sequences, the specific 3′ RACE primers GSP1-GSP2 were designed. Reverse transcription was performed using SMARTScribe™ Reverse Transcriptase and 3′ CDS Primer A. The first round of PCR amplification was performed using GSP1 and UPM primers. The first PCR product was used as the template, and then GSP2 and UPM were used for the second PCR amplification. After electrophoretic detection of PCR products, the target bands were recovered and sequenced.

#### 4.3.3. *Cf*ER Sequence Splicing and ORF Prediction

Finally, according to the splicing results of the 5′ RACE and 3′ RACE, the full-length gene sequence was obtained, and the open reading frame (ORF) prediction was performed. The complete cDNA sequence of *Cf*ER gene was obtained successfully, which laid a foundation for the subsequent functional study.

#### 4.3.4. Tissue-Specific Expression of *Cf*ER

To detect the tissue-specific expression of *Cf*ER genes, total RNA was extracted from five tissues: gonad, gill, adductor muscle, mantle, and digestive cecum of healthy *C. fluminea*. The *β-actin* gene (NCBI login number: EF446608.1) served as the internal reference. The RT-qPCR was conducted followed by data analysis and graphing. The specific sequence of primers required for the experiment is shown in Appendix A Table A4.

### 4.4. Sequence Structure and Phylogenetic Analyses of CfER

Based on the *Cf*ER nucleic acid sequence, the GenBank (https://www.ncbi.nlm.nih.gov/genbank/) (accessed on 10 November 2023) database online website was used to perform BLASTx homology alignment. Then ORF Finder was used to obtain the amino acid sequence of *Cf*ER in the Simple Modular Structure Research Tool (SMART; http://smart.embl-heidelberg.de/) (accessed on 18 November 2023). Signal peptide sequences were further analyzed using the web-based SignalP 4.1 Server (http://www.cbs.dtu.dk/services/SignalP/) (accessed on 20 November 2023) with default d-cutoff and &quot; SignalP-tm&quot; Use ExPASy’s Prot Param server (http://web.expasy.org/protparam/) (accessed on 26 November 2023) and compute pI/Mw server (http://web.expasy.org/compute_pi/) (accessed on 28 November 2023) to characterize the physical and chemical properties of *Cf*ER, including theoretical isoelectric point (pI) and protein molecular mass. The secondary structure of the *Cf*ER protein sequence was analyzed using the Pole Bioinformatique Lyonnais (PBIL) server (https://prabi.ibcp.fr/htm/index.php) (accessed on 2 December 2023).

Using the NCBI database (https://www.ncbi.nlm.nih.gov) (accessed on 10 December 2023) gene homology analysis of genetic data, by selecting the amino acid sequence of *Cf*ER and *Mercenaria mercenaria* ER, *Homo sapiens* ER, *D. rerio* ER, *Aplysia californica* ER, *M. edulis* ER, *A. farreri* ER, *Mizuhopecten yessoensis* ER, *C. gigas* ER, *R. philippinarum* ER, and other sequences, MEGA 11.0 (Institute for genomics and evolutionary medicine (iGEM), Pennsylvania, USA) was used for multiple sequence comparison. For sequence comparison, adjacent-joining trees were constructed using the Poisson correction distance of 1000 bootstrap repeats. The amino acid sequence was analyzed by genetic distance.

### 4.5. Homology Modeling and Molecular Docking of D. rerio and the Three Bivalves

#### 4.5.1. Ligand Preparation and Homology Modeling

In this study, natural estrogen E2 and BPA and its substitutes BPS, BPF, and BPAF were used as study targets. We searched for the above small ligand molecules on ChemSpider|Search (https://www.chemspider.com/) (accessed on 15 December 2023), shared chemistry, and saved the tertiary structure. The three-dimensional structure diagrams of each pollutant are shown in Appendix A Table A1, analyzing the electrostatic potential energy of each pollutant. The registration numbers of target genes obtained through the NCBI website were *D. rerio* ER (NP_694491.1), *A. farreri* ER (ACM16808.1), and *R. philippinarum* ER (QFP12939.1). Three-dimensional modeling of the LBD domain was performed for four species including *Cf*ER. The SWISS MODEL online website Swiss-model (expasy.org) was used to conduct homology modeling for target sequences.

#### 4.5.2. Model Evaluation

This study used discrete optimized protein energy (DOPE) to evaluate the new model coordinates and selected the model with a higher absolute score as a more accurate conformation template. The QMEAN scoring function was used to evaluate the three-dimensional structural plausibility of the target protein model. The QMEAN score range is 4~0. The better the agreement between the model structure and the experiment structure of similar size, the closer the score is to 0, and the built model can be further screened. SAVES v6.0 (https://saves.mbi.ucla.edu/) (accessed on 20 December 2023) was used for PROCHECK and ERRAT scoring to further evaluate the structure.

#### 4.5.3. Molecular Docking

The interaction potential between ERs-LBD and ligands was simulated and analyzed using the AutoDock 4.0 program (The Scripps Research Institute, La Jolla, CA, USA) after selecting the optimal model based on model evaluation criteria. Energy grid calculation was performed using AutoGrid to completely cover the possible active areas. Local Search 4.2 was used to perform local search, set the number of local searches to 50, the number of iterations of the population 150, and select default values for other parameters.

To further explore the binding between the receptor and the ligand, the AutoDock was run for semi-flexible docking, and the most stable ligand-binding mode of the model was determined based on the lowest binding energy (kcal/mol). The docking form and key amino acids were visualized with the PyMol 2.0 plug-in, followed by an analysis of the configuration/score relationship. Image optimization was then carried out using PyMol 2.4.0 software (The Python software foundation, Virginia, USA).

### 4.6. The ERs Gene Expression Under Long-Term Exposure to BPA and Its Substitutes

The *D. rerio* and three bivalves were exposed to BPA and its substitutes BPS, BPF, BPAF, and natural estradiol E2, and blank control and reagent control groups were set up, with three replicates for each group. The exposed concentrations were 0, 1, 10, and 100 μg/L, respectively, and were sampled on days 0, 1, 7, 14, 21, and 28, respectively. Nine individuals were randomly chosen from each group, and their tissues were promptly frozen in liquid nitrogen. Then the samples were stored at −80 °C.

RNA was extracted using Trizol Reagent, Evo M-MLV RT Mix Kit with gDNA Clean for qPCR Ver was used to synthesize cDNA, and SYBR qPCR method was used to perform RT-qPCR to detect the relative expression of ER mRNA in each species. The primer sequence and NCBI login number used in this study are shown in Appendix A Table A4. The ER expression level was analyzed using the 2^−ΔΔCT^ method.

### 4.7. Statistical Analyses

SPSS 26.0 software (SPSS Inc., Chicago, IL, USA) was used to perform all statistics. Visualization was performed using GraphPad Prism 9.0 (GraphPad, San Diego, CA, USA). The homogeneity of variance was tested by Levene. SPSS Statistics was used to statistically analyze all experimental data, employing one-way ANOVA followed by Tukey and Duncan tests. * (*p* < 0.05) were used for significant difference and ** (*p* < 0.001) indicated statistically significant difference.

## 5. Conclusions

In this study, ERs in bivalves were investigated using molecular docking, revealing that BPA and its substitutes bind to ERs via one or more binding sites. Amino acid residues at the binding sites showed similarities to those reported in previous vertebrate studies. Based on the energies during the binding process, as well as the lengths and numbers of hydrogen bonds, it is comprehensively inferred that BPA and BPS have higher affinity and stability with the ERs of bivalves. The expression of ER mRNA in several species in the exposure experiments is highly consistent with the results obtained from the docking analysis, which can mutually validate each other. Finally, the conclusion is that the reproductive toxicity of BPA and BPS is higher than that of BPF and BPA. These BPA substitutes exerted similar effects on the reproductive endocrine system as BPA, posing potential health risks to aquatic animals and warranting serious attention to their toxic effects. This study provides molecular-level references for evaluating binding sites and toxicity risks of typical bisphenol EDCs with bivalve ERs, contributing to the theoretical framework and data support for the monitoring and ecotoxicological assessment of bisphenol pollution in fluvial and marine ecosystems.

## Figures and Tables

**Table 1:** Homology analysis of ERs LBD between *D. rerio*, *C. fluminea*, *A. farreri*, and *R. philippinarum*.

	*D. rerio*	*C. fluminea*	*A. farreri*	*R. philippinarum*
Sequence homology	63.87%	34.25%	36.07%	35.91%
QMEAN score	0.77	0.72	0.70	0.78
DOPE score	−67,057	−60,164	−60,571	−29,680

**Table 2:** Parameter results of model quality check of ERs LBD models for *D. rerio*, *C. fluminea*, *A. farreri*, and *R. philippinarum*.

		*D. rerio*	*C. fluminea*	*A. farreri*	*R. philippinarum*
ERRAT	95% Warning residues (%)	5.4%	0.0%	2.8%	5.2%
	95% Error residue (%)	0.0%	0.0%	0.0%	0.0%
	Quality factor	94.6%	100.0%	97.2%	94.8%
PROCHECK	Core area	92.4%	95.1%	94.1%	93.7%
	General reasonable area	7.6%	4.9%	5.9%	6.3%
	Acceptable region	0.0%	0.0%	0.0%	0.0%
	Unreasonable area	0.0%	0.0%	0.0%	0.0%

**Table 3:** Docking of 3D structures and ligand molecules of ERs LBD with *D. rerio*, *C. fluminea*, *A. farreri*, and *R. philippinarum*.

	Ligand	Ligand Efficiency	Binding Energy	Binding Free Energy	Docked Energy	Final Intermolecular Energy	Inhib Constant	Intermol Energy	Torsional Energy	Unbound System’s Energy	Electrostatic Energy	Involved Amino Acid Residues
*D. rerio*	E2	−0.24	−4.83	−10.27	−10.80	−5.37	286.09	−5.43	0.60	0.07	−0.06	ALA-149
	BPA	−0.19	−3.27	−5.76	−7.46	−3.00	4.03	−4.46	1.19	−0.51	−0.03	PHE-150
	BPS	−0.23	−3.83	−6.90	−8.65	−3.62	1.55	−5.03	1.19	−0.56	−4.98	ALA-149, ALA-121, PHE-152
	BPF	−0.25	−3.81	−6.77	−8.19	−3.19	1.62	−5.00	1.19	−0.23	0.00	PHE-152, SER-128
	BPAF	−0.11	−0.24	−5.31	−7.58	−3.35	16.32	−4.23	1.79	−0.48	−0.05	ASN-163
*C. fluminea*	E2	−0.28	−5.54	−11.68	−12.22	−6.08	87.02	−6.14	0.60	0.06	−0.05	LEU-147, GLN-123
	BPA	−0.09	−1.46	−6.83	−8.47	−5.81	84.76	−2.66	1.19	−0.45	−0.05	GLU-55
	BPS	−0.13	−2.20	−7.00	−8.76	−5.37	24.52	−3.39	1.19	−0.57	−3.32	THR-54
	BPF	−0.10	−1.56	−6.84	−8.21	−5.46	71.95	−2.75	1.19	−0.18	−0.08	LEU-58
	BPAF	−0.07	−1.63	−5.49	−7.9	−4.48	−64.19	−3.42	1.79	−0.62	−0.07	GLN-61, THR-54
*A. farreri*	E2	−0.24	−4.82	−9.89	−10.55	−5.13	293.07	−5.42	0.60	−0.06	−0.29	CYS-67
	BPA	−0.14	−2.43	−5.60	−7.26	−3.63	16.41	−3.63	1.19	−0.47	−3.59	CYS-67
	BPS	−0.25	−4.23	−7.31	−9.08	−3.66	793.52	−5.42	1.19	−0.58	−0.03	LEU-89, SER-36
	BPF	−0.10	−1.49	−4.79	−6.18	−3.50	81.05	−2.68	1.19	−0.20	−0.02	ASN-205
	BPAF	−0.09	−2.10	−5.97	−7.62	−3.73	28.79	−3.89	1.79	0.14	−0.18	ARG-190, GLU-194, TRP-69, MET-218
*R. philippinarum*	E2	−0.18	−3.52	−7.47	−8.13	−4.02	2.65	−4.11	0.60	−0.06	−0.09	ARG-145
	BPA	−0.10	−1.68	−4.31	−6.02	−2.87	58.68	−3.15	1.19	−0.52	−0.09	ARG-177
	BPS	−0.17	−2.84	−7.00	−8.79	−4.04	8.26	−4.75	1.19	−0.60	−0.07	ASN-141
	BPF	−0.09	−1.41	−4.62	−6.1	−2.6	92.59	−3.5	1.19	−0.29	−0.02	ARG-177
	BPAF	−0.06	−1.37	−3.93	−6.27	−3.11	99.68	−3.16	1.79	−0.55	−0.05	LEU-176

## Data Availability

The relevant data of this experiment will be provided proactively if necessary.

## Appendix A

**Table A1:** The composition and 2D structure of the chemical substance required for the experiment.

Chemicals	Molecular Formula	CAS Number	Purity	Chemical Structures
β-Estradiol	C_18_H_24_O_2_	50-28-2	≥97.0%	
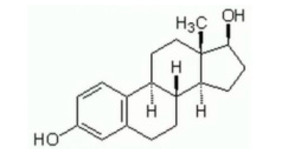
Bisphenol A	(CH_3_)_2_C(C_6_H_4_OH)_2_	80-05-7	≥99.0%	
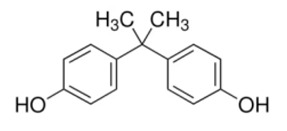
Bisphenol S	O_2_S(C_6_H_4_OH)_2_	80-09-1	≥98.0%	
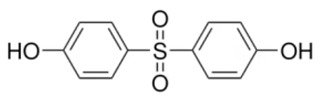
Bisphenol F	CH_2_(C_6_H_4_OH)_2_	620-92-8	≥98.0%	
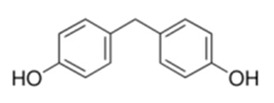
Bisphenol AF	(CF_3_)_2_C(C_6_H_4_OH)_2_	1478-61-1	≥99.0%	
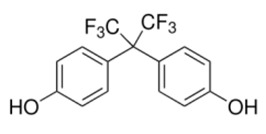
DMSO	(CH_3_)_2_SO	67–68-5	≥99.7%	
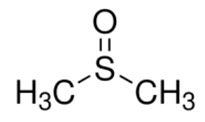

**Table A2:** Aquacultural water quality parameters of experimental animals.

Water Quality Parameter	*D. rerio*	*C. fluminea*	*A. farreri*	*R. philippinarum*
Temperature (°C)	28 ± 1	20 ± 2	18 ± 1	18 ± 1
Salinity (%)	1	1.0	32.0	32.0
pH	7.0	7.0	8.2	8.2
Aquaculture water body (cm^3^)	22 × 16 × 17	22 × 16 × 17	50 × 40 × 30	50 × 40 × 30

**Table A3:** Primers for RACE cloning: 5′ RACE and 3′RACE primers and their sequences.

Genes and Primers Orientation	Primer Name	Sequences (5′-3′)
*C. fluminea* ER	B946-1 (GSP1)	TGCTATCTGCTCCATT
	B946-2 (GSP2)	CCCTGACTCGGATGATCT
	B946-3 (GSP3)	TGTGAAGATGCACCTGATGA
*C. fluminea* ER	C580-1 (GSP1)	GCTATCATAATCATACGCTGCCTC
	C580-2 (GSP2)	TCCAACACGCACACATCTTCTCAC

**Table A4:** RT-qPCR primer sequences of the genes used in the present study.

Genes and Primers Orientation	Sequences (5′-3′)	NCBI Login Number
*Dr*ER-F	ATGTACCCTAAGGAGGAGCA	NP_694491.1
*Dr*ER-R	TCAGGGGTCAGGGCTATG	
*Cf*ER-F	GCCTCCAACACGCACAC	OR365079(unpublished)
*Cf*ER-R	CCATCCAGCAGCACTCA	
*Af*ER-F	ATGTTGATGGGGTGTTTTCAAGTGG	ACM16808.1
*Af*ER-R	TCAATCTCCTTCACTTCCTGTCCG	
*Rp*ER-F	ATGCCTCCACCTAAGAAGCC	QFP12939.1
*Rp*ER-R	CATTAAGACCTGCGCGGTTG	
*Dr*-*β-actin*-F	ACACCCCTGCCATGTATGTG	AF057040.1
*Dr*-*β-actin*-R	GGAAAGCTCTCCCCTGTTAGAC	
*Cf-β-actin*-F	GGCTGTGCTTTCATTGT	EF446608.1
*Cf-β-actin*-R	TTTCTCTTTCGGCTGTT	
*Af-β-actin*-F	CGTGCTCGAGTAGTTGGTGG	KJ081194.1
*Af-β-actin*-R	GTTCTCGAAGTCGAGGGCAA	
*Rp-β-actin*-F	CCAGGCTGTCCTGTCACTTT	EF520696.1
*Rp-β-actin*-R	ACAGTGTGCGATACTCCGTC	
